# Decolonization of carbapenem-resistant *Klebsiella pneumoniae* from the intestinal microbiota of model mice by phages targeting two surface structures

**DOI:** 10.3389/fmicb.2022.877074

**Published:** 2022-08-22

**Authors:** Ju-Yun Liu, Tzu-Lung Lin, Ching-Yu Chiu, Pei-Fang Hsieh, Yi-Tsung Lin, Li-Yin Lai, Jin-Town Wang

**Affiliations:** ^1^Department of Microbiology, College of Medicine, National Taiwan University, Taipei City, Taiwan; ^2^National Laboratory Animal Center, National Applied Research Laboratories Research Institute, Taipei City, Taiwan; ^3^Department of Medical Research, Taipei Veterans General Hospital, Taipei City, Taiwan; ^4^Department of Internal Medicine, National Taiwan University Hospital, Taipei City, Taiwan

**Keywords:** carbapenem-resistant *K. pneumoniae* (CRKP), phage treatment, microbiota, decolonization, receptor

## Abstract

**Background:**

*Klebsiella pneumoniae* is a normal component of the human gastrointestinal tract microbiota. However, in some cases, it can cause disease. Over the past 20 years, the prevalence of antibiotic-resistant bacteria, such as carbapenem-resistant *K. pneumoniae* (CRKP), has been increasing.

**Materials and methods:**

We attempted to specifically eliminate CRKP from a mouse model with the human intestinal microbiota. To establish humanized microbiota-colonized mice, we administered K64 CRKP-containing human microbiota to germ-free mice by fecal microbiota transplantation. Then, we used two phages, one targeting the capsule (φK64-1) and one targeting O1 lipopolysaccharide (φKO1-1) of K64 *K. pneumoniae*, to eliminate CRKP.

**Results:**

In untreated control and φKO1-1-treated K64-colonized mice, no change in CRKP was observed, while in mice treated with φK64-1, a transient reduction was observed. In half of the mice treated with both φKO1-1 and φK64-1, CRKP was undetectable in feces by PCR and culture for 60 days. However, in the other 50% of the mice, *K. pneumoniae* was transiently reduced but recovered 35 days after treatment.

**Conclusion:**

Combination treatment with φK64-1 and φKO1-1 achieved long-term decolonization in 52.3% of mice carrying CRKP. Importantly, the composition of the intestinal microbiota was not altered after phage treatment. Therefore, this strategy may be useful not only for eradicating drug-resistant bacterial species from the intestinal microbiota but also for the treatment of other dysbiosis-associated diseases.

## Background

Phages are viruses that infect bacteria, and lytic phages can lyse and kill bacteria. Therefore, they were used for the treatment of bacterial infections before the discovery of antibiotics. Due to its narrow spectrum, it was not commonly used after antibiotic days. It is now used only for special conditions that are difficult to treat with antibiotics (Kortright et al., 2019). *Klebsiella pneumoniae* is a normal component of the upper respiratory and gastrointestinal tract microbiota in humans (Bowers et al., 2016). However, under some circumstances, it can cause disease. It can cause community-acquired pneumonia, urinary tract infection (UTI), wound infection, and, recently, pyogenic liver abscess (PLA) and necrotizing fasciitis(Fang et al., 2007; Rahim et al., 2019). The increasing prevalence of antibiotic resistance determinants, such as extended-spectrum β-lactamase (ESBL), in *K. pneumoniae*, is a serious problem, and recent global surveillance data from Europe, Asia, and North and South America revealed that the prevalence of ESBL-producing *K. pneumoniae* was 7.5–44% (Reinert et al., 2007). Moreover, the prevalence of carbapenem-resistant *K. pneumoniae* (CRKP) has been dramatically increasing worldwide (Srinivasan and Patel, 2008; Hidron et al., 2009; Sisto et al., 2012; Chiu et al., 2013). Drug-resistant *K. pneumoniae* strains are part of the normal intestinal flora (Bowers et al., 2016), and fecal carriage of CRKP can predispose patients to nosocomial CRKP infections when their immune systems become weakened or impaired (Falagas et al., 2014). Thus, the decolonization of CRKP from the gut of hospitalized patients could prevent CRKP infections (Septimus and Schweizer, 2016). However, antibiotic-mediated decolonization has been reported to be associated with the development of resistance, and selection for resistant Gram-negative organisms (Saidel-Odes et al., 2012; Septimus and Schweizer, 2016). Programmable, sequence-specific antimicrobials using RNA-guided nuclease Cas9 (Gasiunas et al., 2012; Jinek et al., 2012) delivered by a bacteriophage have been developed to reduce a specific strain in microbiota. Both methods reduced the concentration of the target strains *in vitro* and *in vivo*; however, the effects were transient. It has been improved by using phage-delivered CRISPR-Cas9 for strain-specific depletion and genomic deletions in the gut microbiome. However, this method also needs genetic deletion of the target strain besides applying phages (Lam et al., 2021). With the growing knowledge of microbiome, phages are also used to manipulate gut microbiome because its specificity compared with those of antibiotics and cause less change in microbiota. However, the effects are also transient, either by a single phage or multiple phage combinations. An *in vitro* study showed that phage resistance bacteria appeared more frequently if the combination of multiple phages targeting a same receptor (Hesse et al., 2020). In addition, we found isolating phages with wild type *K. pneumoniae* almost all targeting capsules. Therefore, we isolated a phage targeting the lipopolysaccharide (LPS) from a capsule deletion mutant. We chose the most prevent capsule type of CRKP in Taiwan and tried a combination of two phages targeting capsule and LPS in *K. pneumoniae* to eliminate *K. pneumoniae* in intestinal colonization. To mimic the human intestinal microbiome, we also used a humanized microbiota animal by transplanting a patient’s microbiota to germ-free mice.

## Materials and methods

### Human stool collection and isolation and characterization of *Klebsiella pneumoniae* strains

Stool specimens were collected from a patient hospitalized in Taipei Veterans General Hospital with a CRKP infection and CRKP colonization in 2013 (Pan et al., 2013). The *K. pneumoniae* strains present in the specimens were isolated on Eosin Methylene Blue (EMB) agar plates. Their capsular type was determined by *wzc* sequencing (Pan et al., 2013) and confirmed by *wzy* PCR genotyping (Pan et al., 2015). Primer pairs are listed in Table 1. PCR amplifications were performed with the Long and Accurate PCR system (Takara, Tokyo, Japan). The cycling program was 96°C for 3 min, followed by 30 temperature cycles of 96°C for 30 s, 46°C for 15 s, and 72°C for 3 min. Drug susceptibility was assessed using a Vitek 2 system.

**TABLE 1:** Primers used in this study.

Primer name	Sequence	Purpose	Target gene
KP-wzc-CR1	TTCAGCTGGATTTGGTGG	PCR and sequencing	*wzc*
KP-wzc-CR2	GCTTCCATCATTGCAAAATG	PCR and sequencing	*wzc*
K64 wzyF new	CTTTTAGGGCTACGGCACC	PCR genotyping	*wzy*
K64 wzyR new	CCGCGCGCAGG AACATTAG	PCR genotyping	*wzy*
K64 wzy F new	AACAGTTTGCTTATTTCTTGAGTGATG	real-time PCR	*wzy*
K64 wzy R new	AATAACCCAATAAACCCAAATCGA	real-time PCR	*wzy*

Additionally, we tested mice colonized with human microbiota free of *K. pneumoniae* then added K64 CRKP at the beginning. The results were similar. Therefore, the latter model was not used.

### Bacteriophage isolation and preparation

Bacteriophages were isolated from untreated water or soil. The agar overlay method was used for phage isolation, to obtain a pure preparation, and for titer determination. A high-titer phage preparation was generated by amplification and purification using the confluent lysis method as previously described (Lin et al., 2014).

In Taiwan, K64 was the most prevalent capsular type of CRKP (Pan et al., 2015). Therefore, we chose K64 for this decolonization test. To isolate a phage specific for K64 *K. pneumoniae* from the natural environment, a *K. pneumoniae* strain with K64 type capsule was co-inoculated with sewage collected in Taipei City in LB broth overnight. After centrifugation, the supernatant was filtered using a 0.45 μm filter and spotted onto LB plates overlaid with the *K. pneumoniae* K64 strain to detect phage plaques. An agar overlay method was used for the isolation of a pure phage preparation and to determine phage titer. Single plaque isolation, elution, and re-plating were performed repeatedly. The pure isolate of this phage lyzed the K64 type of *K. pneumoniae* strains efficiently and named as φK64-1 (Pan et al., 2017). Capsule type was determined by PCR using primers specific for the K64 wzy gene and confirmed by sequencing. The efficiency of bacterial killing was assessed by using a phage killing assay. Survivors that were resistant to this phage were isolated. Then, the efficiency of bacterial killing by bacteriophage mixtures was examined as previously described (Pan et al., 2017). Actually, all CRKP strains with the K64 capsule type were sensitive to the φK64-1 that was isolated in this study.

We had repeatedly screened phages using wild-type *K. pneumoniae* strains with K1 and K64 capsule types yielded only phages targeting the capsule. To obtain phages that targeted other bacterial surface structures, we constructed a capsule-negative strain by generating a *magA* deletion mutant of NTUH-K2044 on which O1 type LPS was exposed. Screening for phages that infect this mutant yielded a phage, φKO1-1 (Hsieh et al., 2014), that targets O1 LPS.

### *In vitro* phage killing assay

Phage killing assays were done as previously described with minor modifications (Mateus et al., 2014). Briefly, bacteria (1 × 10^4^ CFU) were incubated with phage (1 × 10^5^, 1 × 10^6^, 1 × 10^7^ PFU; i.e., MOI = 10, 10^2^, 10^3^) at 37°C for 30 min. Then, the surviving bacteria were enumerated by plating serial dilutions and determining the CFU, and the survival ratio was calculated.

### Establishment of a humanized intestinal microbiota mouse model

**FIGURE 1:**
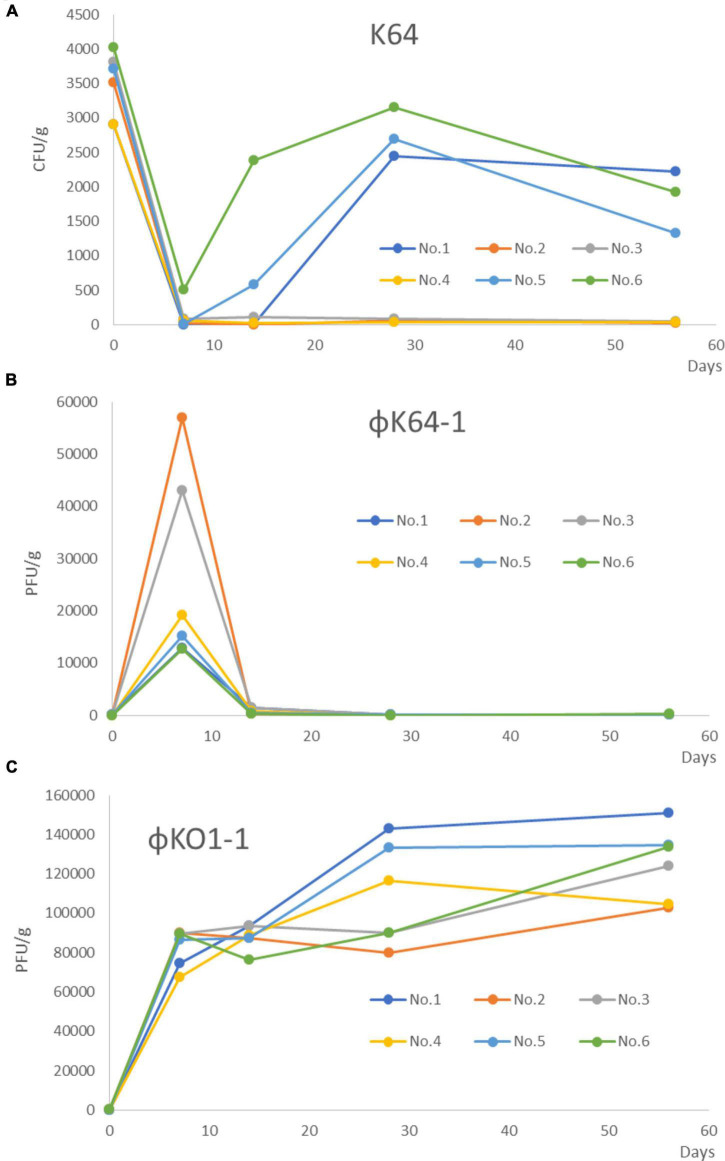
Changed the number of K64, φK64-1, and KO1-1 after treatment with φK64-1 and KO1-1 in the 6 mice colonized with *K. pneumoniae* in the intestines. **(A)** The amount of K64-1 in the mouse intestine. No. 2–4 of the mice in the combination treatment group achieved long-term (56 days) decolonization of K64 CRKP as shown by real-time PCR. No.1, No.5, and No.6 of mice, K. pneumoniae reappeared at 1 week after treatment. **(B)** The amount of φK64-1 in the mouse intestine. The number of φK64-1 reached a peak on the D7, with an average of 26,669 ± 18,717 PFU/g, and then rapidly decreased. **(C)** The amount of φO1-1 in the mouse intestine. No phage was detected from day 0 to 56 in the control group (not shown). The average number of O1-1 on the D7 was 82,976 ± 9,553 PFU/g, and the number continued to rise until day 56. No. 1–6: Mouse number. D: days post phage treatment.

Fecal samples were collected from a patient carrying capsular type K64 CRKP. Then, samples were aliguoted in fresh conditions and stored at -80°C until use. To mimic possible future human inoculations (Sarker et al., 2016), this human-derived fecal microbiota was diluted with saline ten times and orally administered to 7-week-old germ-free C57BL/6 mice (Hong et al., 2016). The colonization of capsular type K64 CRKP in the mice was confirmed by PCR (Table 1) and real-time PCR (Figure 1).

**FIGURE 2:**
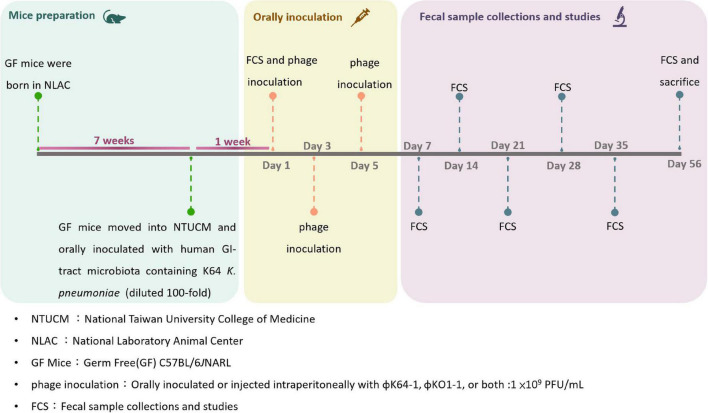
Time course of animal experiments.

The mice were bred and housed in germ-free isolation operation boxes within the animal care facility of the National Taiwan University College of Medicine (NTUCM) and the Laboratory Animal Center at the National Laboratory Animal Center (NLAC). The time course of animal experiments is shown in Figure 2.

### Stool deoxyribonucleic acid extraction, *Klebsiella pneumoniae* detection, and microbiota analysis

DNA was extracted from stool samples using the QIAamp DNA Stool Mini Kit (Qiagen) according to the manufacturer’s protocol. *K. pneumoniae* was detected by PCR, real-time PCR, and confirmed by culture. Real-time PCR amplifications were performed with Power SYBR^®^ Green Master Mix (REF: 4367659). The cycling program was 95°C for 30 s, followed by 45 temperature cycles of 95°C for 5 s and 60°C for 30 s. Primer pairs that targeted *wzy* and *wzc* genes are listed in Table 1. The composition of the microbiota in the established mouse model was determined by the 16s rDNA sequencing. Briefly, the full-length16S gene was amplified by PCR using the V1–V9 region of the 16S rRNA gene primers 27F/1492R (Johnson et al., 2019). Then, the amplicons were purified by using the GeneJET Gel Extraction Kit (Thermo Scientific) and quantified using the Qubit dsDNA HS Assay Kit (Qubit) on a Qubit 2.0 Fluorometer (Qubit). Amplicons were purified using PCR purification kits (Qiagen, Hilden, Germany) and 1 μg of DNA was used for the SMRTbell 1.0 Template Prep Kit (Pacific Biosciences, Menlo Park, CA). SMRTbell-adapted sequences were run on the Pacific Biosciences (PacBio) RS II platform using P6C4v2 chemistry. Consensus sequences longer than 1,600 bp were discarded. *K. pneumoniae* colonization was assessed by PCR, real-time PCR, and confirmed by culture. We tested stool samples at three time points: (1) 1 day before phage inoculation, (2) 14 days after phage inoculation, and (3) 56 days after phage inoculation.

### Phage treatment for the elimination of carbapenem-resistant *Klebsiella pneumoniae* in model mice

Germ-free mice were orally inoculated with human GI-tract microbiota containing K64 *K. pneumoniae* (diluted 100-fold). The mice were divided into five groups 1 week after the models were established: the control group (no phage treatment, group I, *n* = 4); group II (*n* = 4), which was inoculated orally with φK64-1; group III, which was inoculated orally with φKO1-1 (*n* = 4); group IV, which was inoculated orally with both φKO1-1 and φK64-1 (*n* = 28); and group V, which was inoculated orally and injected intraperitoneally with both φKO1-1 and φK64-1 (*n* = 16). The mice were treated with 1 × 10^9^ PFU/mice of bacteriophage (φKO1-1, φK64-1, or both) *via* intragastric injection one time a day for 3 days (day 1, day 3, and day 5). Since mice are coprophilous animals, each mouse was housed in a separate individually ventilated cage (IVC). No cage floors were used in this study. We collected fecal samples just before phage treatment (day 1) and at 7, 14, 21, 28, 35, and 56 days after the first day of phage treatment. DNA isolated from the stool samples was used to detect K64 *K. pneumoniae* by PCR and real-time PCR, and φKO1-1 and φK64-1 were detected by real-time PCR.

### Isolation of carbapenem-resistant *Klebsiella pneumoniae* from mouse feces after 56 days phage treatment and spot test

Carbapenem-resistant *K. pneumoniae* was detected in 13 of the 28 mice at 3–5 weeks after treatment (Table 2). We used the selective medium (DHL agar, Nissui. Code. 05040) with added Ampicillin (100 μg/ml) to isolate the colony (*Enterobacteriaceae*). After the mouse feces were diluted 10 times and 100 times, we used the spread-plate method and incubated at 37°C overnight, and the colonies were selected to confirm whether they were CRKP by PCR. Isolated CRKP (200 μl) were treated with φK64-1 (100 μl) and incubated at 37°C for 15–20 min. The liquid was added into the 3 ml of top agar (LB Broth with 0.7% agar) and was mixed well, before pouring it on LB agar, waiting for the top agar to solidify. It was incubated overnight at 37°C, and the spots were generated.

**TABLE 2:** Intestinal CRKP colonization of mice in each treatment group.

Group (*n*)	Phage treatment		D0	D7	D14	D21	D35	D56
I (4)*	None	*n* = 4	+	+	+	+	+	+
II (4)*	φKO1-1	*n* = 4	+	+	+	+	+	+
III (4)*	φK64-1	*n* = 4	+	-	-	+	+	+
IV (28)*	φKO1-1 and φK64-1 orally	*n* = 11	+	-	-	+	+	+
		*n* = 1	+	-	+	+	+	+
		*n* = 1	+	+	+	+	+	+
		*n* = 15	+	-	-	-	-	-
V (16)*	φKO1-1 and φK64-1 orally and intraperitoneally	*n* = 8	+	-	-	+	+	+
		*n* = 8	+	-	-	-	-	-

CRKP, carbapenem-resistant *K. pneumoniae*.

*Total number of mice in each group.

+, CRKP detected by polymerase chain reaction (PCR).

-, CRKP negative by PCR and culture.

D, days post phage treatment.

## Results

### Isolation of the *Klebsiella pneumoniae* bacteriophages φK64-1 and φKO1-1

The fresh stool specimen of the patient colonized with K64 type *K. pneumoniae* was aliquoted and frozen at -80°C in 2013 until use. The humanized microbiota was established by inoculating diluted frozen aliquoted samples from the patient to germ-free mice (Vandeputte et al., 2017).

The multi-host *K. pneumoniae* bacteriophage φK64-1, which can infect K1, K11, K21, K25, K30, K35, K64, and K69 reference strains, was previously isolated. The genome sequence, which was determined by high-throughput sequencing, was 346,602 bp in length and contained multiple depolymerases (Pan et al., 2017). A second phage, φKO1-1, was isolated that infected acapsular *K. pneumoniae* strains which contain O1 LPS but not capsular strains (Hsieh et al., 2012).

### *In vitro* killing of K64-type *Klebsiella pneumoniae* strains by φK64-1 and φKO1-1

**FIGURE 3:**
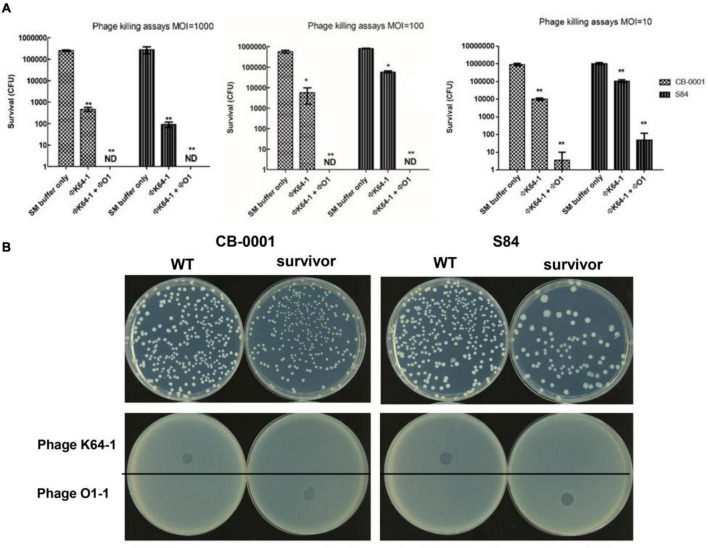
Killing of two K64 *K. pneumoniae* strains by two bacteriophages. **(A)** Killing effects of two bacteriophages in two K64 *K. pneumoniae* strains, CB-0001 and S84. Bacteria were incubated with buffer (control), phage φK64-1, or phages φK64-1 and φKO1-1 (MOI = 10, 100, and 1,000) overnight. Then, bacterial survival was calculated by comparison to the initial counts and is shown as the mean ± standard deviation [SD] from three independent experiments. **p < 0.01 by Student’s *t*-test (compared with control). ND, none detected. **(B)** Phenotype and susceptibility of K64 parental strains and non-capsulated survivors. (Top) Phenotypic differences in the hyper-mucoid phenotypes of the parental and non-capsulated survivor strains on LB agar. (Bottom) Spot tests of phages φK64-1 and φKO1-1 (10^8^ PFU) on the K64 parental and non-capsulated survivor strains.

The killing effects of φK64-1 and φKO1-1 alone and in combination on two K64-type *K. pneumoniae* strains, CB-0001 and S84, were first assessed *in vitro*. At a multiplicity of infection (MOI) of 1,000, 100 and 10, phage φK64-1 killed the majority of the K64 bacteria. The survivors after phage killing (i.e., insensitive to K64-1) were capsule negative strains that formed relatively non-mucoid colonies and were negative for alcian blue staining (data not shown). However, these non-capsulated survivors could be killed by φKO1-1, which targets the O1 antigen of LPS (Fang et al., 2004). Although φKO1-1 could not infect wild-type K64 strains with intact capsule, the combination of φK64-1 and φKO1-1 at MOIs of 1,000 and 100 killed all of the K64 bacteria. However, complete killing achieved only in half of the *in vitro* tests with MOI = 10 (Figure 3A). The survivors after *in vitro* phage killing were all non-capsuled. The wild type was susceptible to phage K64-1, while non-capsuled mutants were resistant to phage K64-1 and sensitive to phage KO1-1 (Figure 3B). The spot tests had been repeated for more than 5 times and showed the same picture.

### Bacteriophage treatment of humanized intestinal microbiota model mice

The *K. pneumoniae* detection assay results by PCR in all study mice are shown in Table 2. *K. pneumoniae* was detected consistently in all untreated humanized microbiota model mice and all model mice treated with φKO1-1 alone. In mice treated with φK64-1 alone, *K. pneumoniae* was undetectable for 2 weeks, but was detected again at 3 weeks after phage treatment. In the 28 mice orally inoculated with both φK64-1 and φKO1-1, *K. pneumoniae* was undetectable from 2 to 4 weeks. However, *K. pneumoniae* was detected in 13 of the 28 mice at 3–5 weeks after treatment. The remaining 15 mice remained *K. pneumoniae*-free through the end of the study (56 days later). Among the 16 mice treated with φK64-1 + φKO1-1 both orally and intraperitoneally, 8 mice showed a transient loss of *K. pneumoniae*, whereas the remaining 8 mice were *K. pneumoniae* negative for 56 days.

The *K. pneumoniae*, φK64-1, and φKO1-1 detection assay results by real-time PCR in 6 mice are shown in Figure 1. The average number of *K. pneumoniae* in all untreated humanized microbial flora model mice was 3,480 ± 473 CFU/g (Figure 1A). Negative real-time PCR results also showed negative PCR results. Therefore, the results of PCR and real-time PCR were consistent.

After the treatment of φ K64-1 and φKO1-1, φK64-1 reached a peak on the 7 days after treatment, with an average of 26,669 ± 18,717 PFU/g, and the average number on the 14 days would drop to 753 ± 538 PFU/g, and it was almost undetectable at 56 days (Figure 1B). However, φO1-1 reached an average of 82,976 ± 9,553 PFU/g on the 7 days after treatment, increased to an average of 87,839 ± 6,224 PFU/g on the 14 days, and continued to increase thereafter, reaching an average of 125,125 ± 18,676 PFU/g at 56days (Figure 1C). No phage was detected from day 0 to 56 in the control group (not shown).

### Changes in the microbiota composition after phage treatment

Results of 16S rDNA sequencing were uploaded to NCBI, PRJNA766775.^1^

The prevalence of *K. pneumoniae* in humanized mice ranged from 0.01 to 0.09% before phage treatments, while the prevalence ranged from 0.04 to 0.08% 56 days after failed treatments.

**FIGURE 4:**
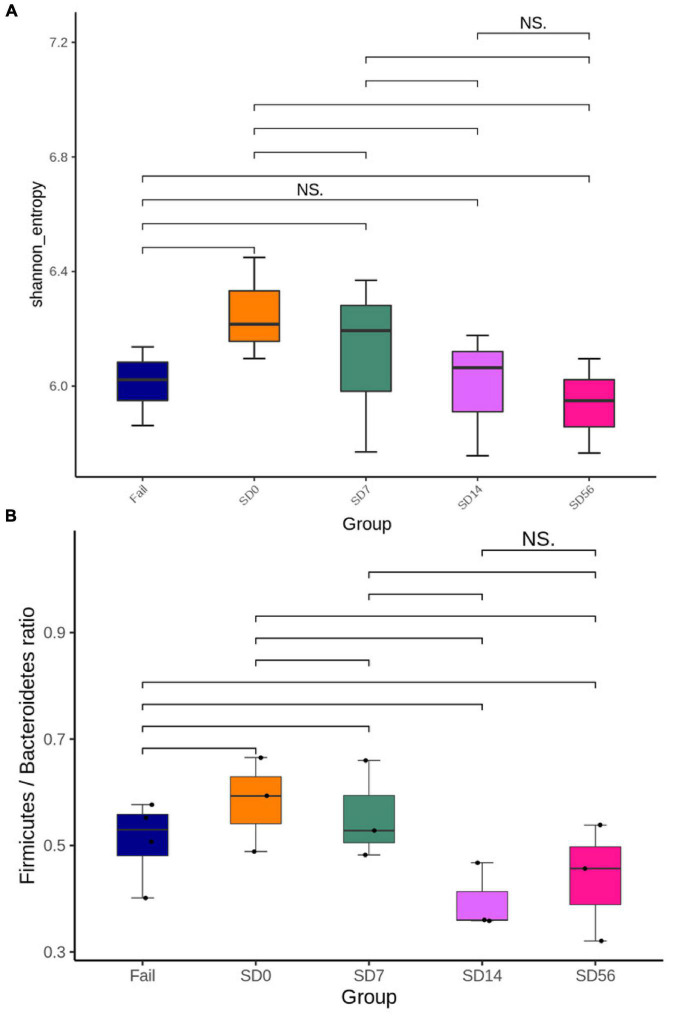
Changes in the intestinal microbiota composition before and after treatment with phages φK64-1 and φKO1-1 for the removal of CRKP. **(A)** Shannon’s diversity index. SD0 had the highest species diversity, and then species diversity gradually decreases. There was no significant difference between the SD14 and Fail groups. **(B)** Firmicutes/Bacteroidetes ratio. The F/B ratio of Fail, SD0, and SD7 groups were greater than 0.5, and there was no significant difference between the SD14 and SD56 groups. S: CRKP cannot be detected by PCR after phage treatment. Fail: CRKP detected by PCR after phage treatment. D: days post phage treatment.

Shannon’s diversity index is commonly used to characterize species diversity in a community. Germ-free mice were orally inoculated with human GI-tract microbiota, so Shannon’s diversity index was highest before treatment (SD0). The difference then become lower at SD14 and SD56 (Figure 4A).

The Firmicutes/Bacteroidetes (F/B) ratio is widely accepted to have an important influence in maintaining normal intestinal homeostasis. Increased or decreased F/B ratio is regarded as dysbiosis, whereby the former is usually observed with obesity, and the latter with inflammatory bowel disease (IBD; Stojanov et al., 2020). The F/B ratio was the highest before phage treatment (SD0). However, there was no significant difference between SD14 and SD56, and it fell below 0.5 at D56, indicating that the GI-tract microbiota might gradually return to balance (Figure 4B).

### Spot tests for carbapenem-resistant *Klebsiella pneumoniae* survived after combination phage treatments

**FIGURE 5:**
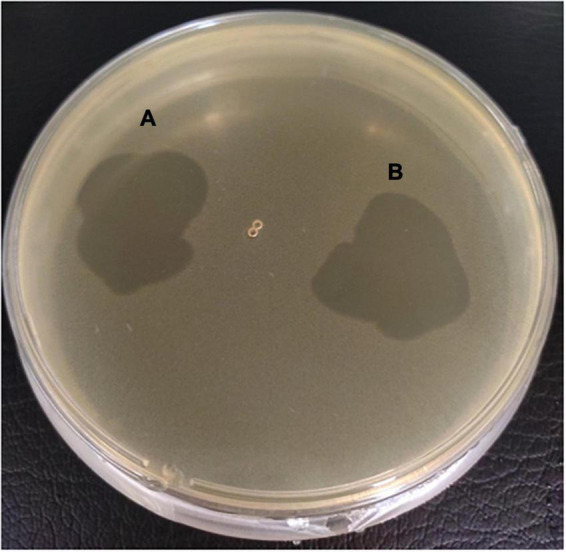
The spot test of isolated CRKP from mouse feces. The concentration of φK64-1 were 1 × 10^5^ **(A)** and 1 × 10^6^ **(B)** PFU/ml.

Carbapenem-resistant *K. pneumoniae* were isolated from mouse feces after 56 days of phage treatment failed mice, and were confirmed as K64 by PCR (data not shown). We observed that, in contract to the *in vitro* killing assays, the isolated CRKP could still be infected by φK64-1 by plaque assay (Figures 5A,B). The amount of φK64-1 was the highest after 7 days of phage treatment, but it was decreased by more than 1 × 10^4^ times after 14 days of phage treatment (Figure 1B).

## Discussion

Our previous survey revealed that K64 was the most prevalent CRKP type in Taiwan; therefore, we chose K64 type *K. pneumoniae* as the model strain in this study. Our results showed that treatment with phage φK64-1 only transiently suppressed the K64 strains. This result agreed with previous findings in phage treatment studies. However, 52.3% of the mice in the combination treatment group achieved long-term (56 days) decolonization of K64 CRKP as shown by PCR and culture. In the other 47.7% of mice, *K. pneumoniae* reappeared at 3 weeks after treatment, indicating transient suppression (for about 2 weeks), which was similar to that observed with the φK64-1 treatment alone. A recent study found that the administration of a cocktail of three bacteriophages to LF82-colonized CEABAC10 transgenic mice significantly but transiently (for several weeks) decreased the number of adherent-invasive *Escherichia coli* (AIEC) in feces and the adherent flora of intestinal sections (Galtier et al., 2017). In our *in vitro* assay, capsule-negative mutant strains appeared immediately after φK64-1 treatment. Since phage screening using wild-type *K. pneumoniae* only identified phages targeting capsules (Hsieh et al., 2012; Pan et al., 2017), combinations of naturally selected phages fail to kill mutants with a single outer surface change.

Although fecal microbiota transplantation has been used to successfully eradicate drug-resistant strains (Davido et al., 2017; Feehan and Garcia-Diaz, 2020), the potential side effects make this method less satisfactory. In addition, the procedures are more tedious than combination phage therapy. Failure of the combination treatment may be due to an inadequate of φK64-1 dosage. In our *in vitro* studies, no mutants resistant to both φKO1-1 and φK64-1 were detected. However, long-term clearance of CRKP was not achieved in half of the mice. Phage K64-1 is specific to K64, so when K64 was reduced and phage K64-1 decrease rapidly (Figure 1B). It may be the reason why K64 cannot be completely eliminated. O1 polysaccharides in GI-tract microbiota will be recognized by phage O1, so the number of phage O1 was still maintained (Figure 1C). In two mice that failed to colonize, CRKP detected in 1 or 2 weeks (Figure 1A). Quantification by 16S rDNA, Proteobacteria decreased by about 50% after phage treatment and remained stable until day 56. The titers of φK64-1 were very low by real-time PCR. It may be caused by experimental procedures or individual mice difference. This may be due to the limited stomach volume of mice, which allowed a maximum dose of 1 × 10^9^ PFU by oral inoculation. If this is the case, oral inoculation for more than 3 days may achieve a higher decolonization rate in mice. In human, higher doses of inoculation may also achieve higher successful rates.

One side effect of using antibiotic treatment to suppress a bacterial species in the intestine is alteration of the microbiota composition. The combination phage treatment used in our study seemed to cause fewer changes in the microbiota during and after treatment, and the F/B ratio seemed generally returned to pre-treatment status by 2 months after treatment. This suggests that this strategy not only has the potential to be used to reduce or eliminate drug-resistant *Enterobacteriaceae* but also has a great potential for the treatment of other bacterial species associated with dysbiosis and systemic-related diseases.

In conclusion, treatment with the combination of two bacteriophages, φK64-1 and φKO1-1, achieved long-term decolonization of CRKP in 52.3% of model mice carrying CRKP. The intestinal microbiota largely was not altered by the phage treatment. These data indicate that this strategy may be useful for not only eradicating drug-resistant bacterial species from the intestinal microbiota but also for the treatment of dysbiosis-associated diseases.

## Data availability statement

The datasets presented in this study can be found in online repositories. The names of the repository/repositories and accession number(s) can be found in the article/supplementary material.

## Author contributions

J-TW and J-YL designed the study, analyzed the data, and revised the manuscript. J-YL, C-YC, and Y-TL prepared materials and performed the experiments. J-YL, T-LL, and P-FH analyzed the data. J-YL, T-LL, L-YL, and P-FH wrote the main text of the manuscript. All the authors reviewed and approved the final version of the manuscript.

## Footnotes

1
https://www.ncbi.nlm.nih.gov/sra/PRJNA766775
